# Hospital costs of extracorporeal life support therapy

**DOI:** 10.1186/2197-425X-3-S1-A947

**Published:** 2015-10-01

**Authors:** WM van den Bergh, Dutch ECLS Study Group

**Affiliations:** Critical Care, University Medical Center Groningen, Groningen, Netherlands

## Introduction

Extracorporeal life support (ECLS) seems an efficient therapy for acute, potentially reversible cardiac or respiratory failure, when conventional therapy has been inadequate, or as bridge to transplant, but there is no evidence for cost-effectiveness. Our aim was to conduct an exploration of the hospital costs of ECLS therapy.

## Objectives

Single-center retrospective exploratory cohort cost study. The study is performed from a hospital perspective with a time horizon of patients' complete hospital admission in which they received ECLS.

## Methods

All 67 consecutive adult patients who were admitted to the ICU of the University Medical Center Groningen (the Netherlands) in the period 2010 to 2013 and received ECLS treatment were included. The bottom-up micro costing method was used. Medical costs were estimated by multiplying every registered healthcare consumption with unit prices. Unit prices were largely based on Dutch reference prices. For each patient the personnel costs and material costs were assessed in detail. The costs of ECLS were differentiated in costs of procedures and costs of daily surcharge of therapy. Procedure related costs were subdivided in costs of devices and disposables, costs of additional human resources and surgery hours.

In order to estimate costs of ECLS therapy based on indication all patients were categorized into six different subgroups: respiratory - bridge to recovery, respiratory - bridge to transplant, cardiac - bridge to recovery, cardiac - bridge to transplant, cardiac - post-cardiotomy, and ECPR.

## Results

The mean age was 46 years, female sex 66%, APACHE II score 24, ECLS duration 135 ± 163 hours, ICU length of stay 18 ± 22, hospital length of stay 38 ± 45, and in hospital mortality 68%. The mean total hospital costs were €106.263 (€83.841 - €126.266) per patient. On average, 52% of the total costs arose from hospital nursing days and 11% of direct procedure related ECLS costs. Surgery and diagnostics represented a vast amount of the remaining costs.

**Table 1:** Table 1

Patient category	Mean total hospital cost (€)	Mean cost per hospital day (€)
All patients	106.263	2,796
respiratory - bridge to recovery	110,553	2,571
respiratory - bridge to transplant	153,345	2,513
cardiac - bridge to recovery	69,803	5,369
cardiac - bridge to bridge/transplant	66,971	2,480
cardiac - post cardiotomy	68,582	5,275
ECPR	51,997	3,999

*[Hospital cost per category]*

## Conclusions

This large and detailed economic evaluation of hospital costs of ECLS therapy in the Netherlands showed that mean total hospital cost of ECLS treatment are €106.263 per patient. The majority of the costs are composed of hospital nursing days.

